# Economics of Philanthropy

**Published:** 1898-05-14

**Authors:** 


### The Institutional Workshop.

### ECONOMICS OF PHILANTHROPY.

### VII.—ORPHANS.

There is always something pitiful in the sight of an orphan. The more fully one accepts the idea of the divine origin and intention of the family, the sadder does it appear when the children are deprived of those who should in the ordinary course of things have maintained and protected them. Even a rich orphan is not a creature to be envied; and a poor one, left to the tender mercies of relations, is often one of the most pitiable of creatures. He has done nothing to deserve his loneliness, his isolation, his dependence; nor can we blame his parents altogether. While it is well for a man to make some provision for his children, even before they are born, it is practically impossible that everyone should do so. The ordinary expectation of every man is that he will live to see his children grow up to an age at which they will be self-supporting. This is the ordinary expectation and the ordinary result, and the cases in which this expectation fails are so few as to merit exceptional treatment. That this is generally felt may be assumed from the fact that almost every occupation has an orphan asylum or an orphan fund, by which the children of deceased fellow-workers may be supported. On a larger scale, and without reference to the original position of the parents, such homes as Barnardo's, Quarrier's, and Muller's take in orphans, educate them, teach them useful trades, and often in the end send them abroad.

The question for us, in this inquiry, is to what extent these institutions are economically justified, and on what lines they are best developed? It is hardly necessary to say that any plan which brings up children to be useful citizens is good. Preferably, one would wish that the burden of so bringing them up should be borne by those who gave them life; but this would involve conditions which are not always possible. Taking things as we find them, we accept the fact that some children of every class will have to be provided for by others than their parents. The guardians of the poor throughout the country will always conduct the largest orphanages; but various advantages will be obtained by those who can claim admission to institutions of a more private character. The real difficulty in all such institutions is, not to find the money to carry them on—in a rich and generous community like ours that is easy enough—but how to use it in such a way as to produce good citizens. Home life, including free, yet regulated, intercourse with the world, being the best for any child, the question comes to be to what extent institutional life can imitate this and give as good a training for maturity? There is a strong feeling among many that merely to feed, clothe, and educate orphan children does not succeed in this. Institutions based on the model of the convent do not always show good results, if the after-career of the children brought up in them be taken into consideration. The city of Edinburgh, which is peculiarly rich in educational foundations, took a step about thirty years ago which showed a belief that orphan asylums of the old school are not the most useful to the beneficiaries. The governors of several of these—Watson's, Stewart's, Gillespie's, and the Merchant Maiden's Hospitals—abolished their homes and conventual institutions, and with their funds created schools, some free, some at which the fees are exceedingly small, considering the quality of the education given, which is of the best. By this means many outside the ranks of the original beneficiaries profit; but it is considered that these beneficiaries are also better off. Those who under the old *régime* would have been eligible for residence in the hospitals, are now boarded out with their mothers, if the mothers are alive, while others are placed in respectable families, chosen by the governors of the Merchant Company, in whom the patronage of the various charities was vested. They attend school at the seminaries of the associated charities. Thus, whenever practicable, home life is preserved, the mothers receiving payment for the maintenance of their children, who under other circumstances would have been removed from the family circle, and placed in the orphanage. The change was made because the governors in charge of the various funds were convinced that the conventual system was bad for the children. Boys who had grown up in an atmosphere which was artificial, however good it might be, were, it was found, less fit to meet the contingencies of life than those who had spent their childhood in a wider sphere. They were easily cheated, easily led astray. With regard to the girl pupils of the "Merchant Maidens' Hospital," the results were often even worse. The statements as to the number of those who recruited the class of fallen women might be, and probably were exaggerated, but that such reports should be current implied that something was wrong with the method of education. The truth is, that knowledge of the world, of the rough and the smooth, the dangers and pitfalls as well as the honourable openings to success, are as essential a part of education as any amount of Latin, Greek, French, and science. It was in this part of education that these conventual institutions failed, and it was for this reason that the system was abandoned. There may have been some complaints made about the new system, chiefly by those teachers in private schools who have found that the lower fees and equal, if not superior, advantages of the endowed schools emptied their establishments; but there has never been any serious recommendation to return to the old system.

The moral of all this is obvious. Orphan funds are necessary, and we can foresee no future when they shall be no longer needed; but the administration of them in many cases leaves a good deal to be desired. They do not succeed in their object if they do not prepare their beneficiaries for life in the largest sense. Food, clothing, shelter, education are all essential; but the orphan asylum does not fill the place of the home unless it gives also lessons in self-protection and knowledge of the world. This is the most difficult of all its tasks; and when visitors to an orphanage are impressed by the handsome building, the admirable behaviour of the orphans, the good food and excellent education provided for them, it is worth while to ask if this impalpable but supreme essential is added to all these. For our own part, we are of opinion that, where it is possible, a child should be kept at home, and the surviving parent paid for its maintenance; and, in other cases, the governors should try to board the children out in suitable families, not necessarily in one neighbourhood, and let them attend school somewhere near their temporary home. This would involve some extra work, and would certainly mean less show of the kind that draws subscriptions out of the pocket of the visitor to the orphanage, but we think it would be better for the orphans themselves.

In connection with orphanages, one is almost compelled to speak of those societies which assist emigration, as many orphanages and other shelters for homeless children send their *protégés* abroad. We think that these societies do a most useful work. Speaking from experience of Canada, we are prepared to say that these children—orphans, friendless, often foundlings—have opportunities there which they would not otherwise obtain of becoming useful and self-supporting citizens; and some of them have risen to positions of distinction. Every care seems to be taken to place out the children in families where they will be well treated; and in childless homes, or even where the children are grown up, they often become members of the family. Occasional instances of harsh treatment may doubtless be found; but, on the whole, the fate of these children who are thus emigrated is better than it would have been at home, often incomparably better. In our colonies there is still so much space that those who go there are not afflicted, as some of the weaker brethren are here, with the notion that they are not wanted in this world. If their present situation does not suit them they look for another. Some succeed, some fail, as happens all the world over; but none are hindered from making an effort from the notion that there is none possible for them to make. The moral atmosphere of new countries is invigorating, and those who have least reason for clinging to the old are often the most successful there.

Occasionally, indeed, one hears complaints from the colonies that their soil is made the "dumping-ground" of the failures of the old world; that we send them children, the offspring of worthless or criminal parents, whose hereditary habits come out in after life, and add to the vagrant and criminal population of the country of their adoption. While allowing for the persistence of tendencies, especially bad ones, that are "bred in the bone," we cannot but think that these complaints are for the most part unfounded. In this connection we are justified in quoting the opinion of a Canadian judge, Mr. Henry, as expressed in a reply to the Grand Jury of Hamilton, Ontario. The jury, incited by the Labour Party, who feared that an increase in the number of labourers might lower wages, stated that crime was increasing in Canada, and expressed their belief that the introduction of the English children was the cause. "Gentlemen," said the judge, "crime is increasing in Canada. But it is not the English children. In a long experience I have had only *two* brought before me. I will tell you what it is that is the cause of the increase of crime; *it is your godless system of education.*" (We quote from a letter of Miss Maria Rye, through whom 4,000 children have been emigrated to Canada. The italics are hers.)

But to one form of asylum for children we must express a strong objection, the Foundling Hospital. The effect of such institutions is almost wholly evil. They enable, and as conducted in some parts of the Continent, absolutely encourage parents to evade their natural responsibilities. Sentimental writers draw pitiful pictures of the tears of the mother, as she leaves her child at the door of the hospital. In some cases the parting may cost a pang; but the fact remains that the child is deserted; the parents, one or both, not only delegate their natural duty to others, but choose to know nothing of the future of their offspring. The effect on the morals of the community of encouraging such evasion of duty does not need to be dwelt on; and, on the other hand, the records of foundling hospitals, especially abroad, often show such a mortality as to indicate that there is a feeling even on the part of those who have undertaken the care of these "nobody's children," that they are not wanted in the world at all.

